# Impact of poor-quality medicines in the ‘developing’ world

**DOI:** 10.1016/j.tips.2009.11.005

**Published:** 2010-03

**Authors:** Paul N. Newton, Michael D. Green, Facundo M. Fernández

**Affiliations:** 1 Wellcome Trust–Mahosot Hospital–Oxford Tropical Medicine Research Collaboration, Microbiology Laboratory, Mahosot Hospital, Vientiane, Lao PDR; 2 Centre for Clinical Vaccinology and Tropical Medicine, Churchill Hospital, University of Oxford, Oxford, OX3 7LJ, UK; 3 Division of Parasitic Diseases, Centers for Disease Control and Prevention, Atlanta, Georgia, 30333, USA; 4 School of Chemistry and Biochemistry, Georgia Institute of Technology, Atlanta, Georgia, GA 30332-0400, USA

## Abstract

Since our ancestors began trading several millennia ago, counterfeit and substandard medicines have been a recurring problem, with history punctuated by crises in the supply of anti-microbials, such as fake cinchona bark in the 1600s and fake quinine in the 1800s. Unfortunately this problem persists, in particular afflicting unsuspecting patients in ‘developing’ countries. Poor-quality drugs are a vital (but neglected) public health problem. They contribute to a ‘crevasse’ between the enormous effort in therapeutic research and policy decisions and implementation of good-quality medicines.

## Introduction

Globalization of the pharmaceutical industry has the potential to rapidly spread poor-quality medicines worldwide before adequate detection and intervention are possible. There are two main categories of poor-quality medicines: substandard and counterfeit (Box 1). Substandard products arise as a result of lack of expertise, poor manufacturing practices, or insufficient infrastructure, whereas counterfeits are the ‘products’ of criminals [1,2]. Counterfeits may contain no active ingredient, incorrect ingredients, or toxins. The amount of active ingredient does not provide sufficient information to accurately determine if a medicine is counterfeit; inspection of the packaging is also required as mislabelling is a key part of the definition and counterfeits with fake packaging but the correct amount of active ingredient have been described (Box 1). In many reports, it is unclear if poor-quality medicines are counterfeit or substandard, but it is important that they are correctly classified because they have different origins and different solutions. Inadequate enforcement, lenient penalties, corruption, ‘spaghetti-like’ trade arrangements, unregistered medicines, and ignorance of poor-quality medicines among the public and health workers exacerbate the situation [1–3].

## Prevalence

There are very few published data allowing estimation of the extent of the problem and the impact on public health [1–7]. Only 5–15% of the 191 member states of the World Health Organization (WHO) report cases of counterfeit drugs [2]. Many data have been interpreted uncritically; some are inaccurate and do not allow accurate generalizations about the epidemiology of poor-quality medicines [1–7].

Counterfeits of most commonly used essential drugs have been described, with a recent review describing 206 cases of counterfeit anti-infectives from 38 countries [2]. Of 771 reports of counterfeit medicines received by the WHO from 1982 to 1999, 48.4% were from the Western Pacific region, with most being labelled as anti-infectives [3]. The International Medical Products Anti-Counterfeiting Taskforce (IMPACT) cautioned against using the off-quoted estimate of 10% of the global supply being counterfeitt, and suggested that “many developing countries of Africa, parts of Asia, and parts of Latin America have areas where >30% of the medicines on sale can be counterfeit. Other developing markets, however, have <10%…”[5].

Anti-malarials appear to have been particularly targeted. In a recent epidemic of fake artesunate in mainland South-East Asia 38%–53% of these vital anti-malarials obtained from pharmacies and shops were counterfeit, revealing a wide diversity of counterfeit packaging types [2,8]. After hundreds of patients with visceral leishmaniasis failed to respond to ‘miltefosine’ in Bangladesh, capsules were found not to contain miltefosine [9]. Diagnostic tests have also been faked, including counterfeit lactate test strips and HIV antibody kits. Counterfeit insecticide-treated bednets and vaccines (against e.g. *Neisseria meningtidis*, influenza and rabies) are of considerable concern because of their importance in preventing key diseases [2]. With intimate links between the health of humans and animals, poor-quality insecticides and veterinary medicines suggests unappreciated interrelated consequences for the health of livestock and humans.

Substandard products have also been with us since medicines were first compounded. They are an inevitable consequence of inadequate local regulation of the pharmaceutical industry and the lack of good manufacturing practices (GMP) facilities in many ‘developing’ countries [1,9]. In Venezuela, for example, primaquine tablets were found to contain 19–168% primaquine, and one patient developed *Plasmodium vivax* malaria after taking primaquine containing 46% of the stated content [2]. A significant proportion of sulphadoxine–pyrimethamine tablets available in Africa are substandard and fail dissolution testing because of incorrect formulation, resulting in poor oral bioavailability and reduced efficacy [2].

## Impact

Considering the vast scale of the global pharmaceutical industry and the incidence of potentially fatal diseases, any amount of poor-quality medicine is unacceptable because it increases morbidity and mortality (Box 2). The impact of poor-quality medicines is most clearly evident if they contain lethal incorrect active ingredients. Until recently, it was often assumed that counterfeits were inert. However, forensic chemistry has demonstrated that many contain harmful ingredients – as tragically illustrated by the death of ∼500 children after ingesting paracetamol containing a renal toxin [2]. Patients may also suffer adverse effects of unexpected ingredients, e.g. co-trimoxazole containing diazepam; reused ceftazidime vials containing streptomycin; and counterfeit artesunate tablets containing artemisinin, chloramphenicol, paracetamol, and metamizole. Patients may be allergic to these covert pharmaceuticals, or may experience confusing adverse events. Some substandard drugs contain more active ingredient than stated [10] and, for anti-infectives with narrow therapeutic ratios, this may increase the prevalence of adverse effects.

The use of counterfeit anti-malarials, and the consequent failure of patients to improve, has led to false reports of drug resistance to malaria [13]. An example of the potential dangers of sub-therapeutic dosage were illustrated when heavier tourists, dosed without taking patient body weight into account, and not their thinner co-travelers, developed *P. vivax* relapses [11]. Anti-infectives containing sub-therapeutic amounts of the active ingredient (whether counterfeit or substandard) increase the risk of the selection and spread of drug-resistant pathogens [13]. Selection depends on a wide variety of factors, i.e. pathogen biomass; host immunity; relationships between the drug pharmacokinetic profile; pharmacodynamic effects on the pathogen; anti-microbial susceptibility of the the pathogen; and the fitness of resistant mutants. If resistant pathogens infect or arise *de novo* within a host and encounter sub-lethal concentrations of a slowly eliminating anti-microbial, they will have a survival advantage and multiply faster than sensitive pathogens [12]. Although models of the emergence and spread of resistance to anti-malarial drugs suggest that poor-quality drugs are important, it is very difficult to tease apart the effects of the misuse of anti-infectives by health workers, patient adherence, and poor-quality drugs. Counterfeits containing no active ingredient will not provide this ‘drug pressure’, and it is likely that substandard medicines are more important in engendering resistance. However, fakes containing sub-therapeutic amounts of the stated ingredient, or incorrect anti-microbial ingredients, may facilitate the emergence and spread of drug-resistant pathogens. For diseases treated with combination therapy (e.g. tuberculosis, HIV, falciparum malaria), poor-quality combination medicines risk the spread of resistance due to the poor-quality active ingredient and the ‘unprotected’ co-ingredient. Artemisinin derivatives-based combination therapies (ACTs) hold great hope for controlling malaria in Africa but, most alarmingly, poor-quality ACTs are already widespread [2,6,13]. *Plasmodium falciparum* artesunate resistance has recently been described on the Thailand–Cambodia border and the wide use of monotherapy, substandard artesunate, and fake artesunate containing sub-therapeutic quantities of artemisinin and artesunate in South-East Asia have probably contributed to this potentially disastrous problem [8]. Poor-quality tuberculosis (TB) drugs [14] are a neglected link between TB treatment, therapeutic failure and the increasing burden of TB drug resistance.

## Combating the problem

Strengthening medicine regulatory authorities (MRAs), improving quality of production, and facilitating the availability of relatively inexpensive, good-quality anti-infectives are likely to be key factors in improving drug quality. There is an urgent need for data of sufficient sample size with random sampling design to reliably estimate the prevalence of poor-quality medicines. Such data are vital to select appropriate interventions, assess their effectiveness, and follow changes through time. Recent literature has concentrated on poor-quality anti-malarials, but it is likely that other anti-infective medicines are also profoundly affected. We do not know how counterfeit and substandard medicines compare with respect to their impact. We also have little information on what proportion of patients or health workers are aware of the issues in different societies, and which interventions may be the most effective.

Sustained political will and financial support for coordinated action from the police, customs officials and MRAs is crucial. A recent forensic investigation into the trade in fake artesunate demonstrated that police, scientists, the pharmaceutical industry, governments and the WHO can work together to combat these problems [8]. Although a wide range of sophisticated quality-assurance markers have been developed, they are unlikely to be implemented in the poorest countries. A major limitation is MRA capacity; WHO estimated that 30% of countries have no drug regulation or a capacity that hardly functions [2,4,7]. The lack of financial and human resources available to many MRAs makes investigation of poor-quality drugs and action impossible. There are only two WHO pre-qualified quality-control medicine analysis laboratories in the whole of malarious parts of Africa [15]. Support for MRAs and the development of regional laboratories to allow the regulation of the drug supply will be crucial to allow interventions. The actions necessary to combat substandard drugs may be more straightforward because criminal deception is not involved, but these interventions will involve costly improvements in GMP and periodical inspections. Increased provision of free or inexpensive medicines for key diseases would undercut the counterfeiters and reduce the criminal financial incentive. The available evidence suggests that poor-quality essential medicines are having a very important (but avoidable) toll on health in the developing world, and that this issue clearly needs to be taken much more seriously. We remain woefully ignorant as to how these problems can be addressed.

## Conflict of Interest Statement

None of the authors have a conflict of interest.

## Box 1 Definitions and examples of poor-quality medicines [1–3,7]

**Counterfeits** are “deliberately and fraudulently mislabelled with respect to identity and/or source. Counterfeiting can apply to branded and generic products. Counterfeits may include products with correct or incorrect ingredients, without active ingredients, with insufficient active ingredient, or with fake packaging” [3,7]. For example, an antidepressant (fluvoxamine) and a muscle relaxant (cyclobenzaprine hydrochloride) were labelled as anti-retrovirals in the Democratic Republic of Congo. One-third to one-half of the a vital anti-malarial agent artesunate bought in mainland South-East Asia is counterfeit, containing no or sub-therapeutic active ingredient
**Substandard drugs** are “genuine drug products which do not meet the quality specifications set for them” [3,7]. For example, contaminated substandard magnesium sulfate leading to *Serratia* septicaemia; 10% of anti-TB medicines contained less than allowed active ingredient in six TB-endemic countries; and gentamicin-resistant *Pseudomonas aeruginosa* in gentamicin eye drops led to severe eye infections.

## Box 2 Impact of poor-quality medicines

• Increased mortality and morbidity
• Engendering of drug resistance and loss of medicine efficacy
• Loss of confidence in health systems and health workers
• Economic loss for patients, their families, health systems, and the producers and traders in good-quality medicines
• Adverse effects from incorrect active ingredients
• Waste of enormous human effort and financial outlay in development of medicines, optimising dosage, carrying out clinical trials, discussing policy change, and manufacturing medicines
• Increased burden for health workers, medicine regulatory authorities (MRAs), customs officials and police officers
